# A reassessment of existing systematic reviews evaluating the effectiveness of cryotherapy in patients following total knee arthroplasty

**DOI:** 10.1080/07853890.2025.2512432

**Published:** 2025-06-04

**Authors:** Rui-Hong Niu, Xue-Feng Hou, Feng Chang, Ru Wang, Ze-Hua Qu, Peng-Guo Gou

**Affiliations:** Department of Orthopedic Surgery, Shanxi Provincial People’s Hospital Affiliated to Shanxi Medical University, Taiyuan, Shanxi, China

**Keywords:** Cryotherapy, total knee arthroplasty, overviews of reviews

## Abstract

**Objective:**

This study reassessed systematic reviews on cryotherapy efficacy following total knee arthroplasty (TKA).

**Methods:**

Ten databases were searched from January 1, 2018 to September 15, 2023 for relevant systematic reviews. Two researchers independently screened articles and extracted data.

**Results:**

Five systematic reviews were included. Two of the studies were found to exhibit high methodological quality, while the remaining three demonstrated exceedingly low methodological quality. The deficiencies in quality were attributed to several factors, including the lack of a predefined research protocol, absence of exclusion criteria, unclarified funding sources for the included studies, and potential for publication bias. Of the 18 outcome indicators, Swelling was intermediate-quality evidence , Hemoglobin, Pain score within 24 hours after surgery, Pain score within 48 hours after surgery, Pain score within 72 hours after surgery, Blood loss volume, Pain score on the next day of surgery and Range of Motion (ROM) were low-quality evidence, and other outcome indicators were exceedingly low-quality evidence.

**Conclusion:**

Cryotherapy appears to offer benefits such as pain relief, reduction in joint swelling, and improved joint mobility during the initial post-operative phase following TKA. However, further high-quality studies are required to validate the efficacy and safety of cryotherapy for patients undergoing TKA, given the identified limitations in the quality of the available outcome indicators.

## 1. Introduction

Total knee arthroplasty (TKA) is one of the most common procedures performed in orthopedic surgery [1], and is highly effective in alleviating pain and enhancing joint function for patients with severe arthritis [2]. Following TKA, patients are encouraged to commence rehabilitation promptly to restore knee joint functionality and muscle strength [3]. Nevertheless, approximately 20% of patients who undergo TKA experience persistent or recurring pain [4], as well as other adverse events such as swelling, inflammation, and bleeding [5], significantly hindering their rehabilitation progress [6].

Cryotherapy, a non-pharmacological treatment approach [7], is a potential option in postoperative rehabilitation [8]. By lowering tissue temperature, constricting blood vessels, and decreasing blood flow, cryotherapy effectively alleviates pain, reduces swelling, and accelerates recovery [9]. Commonly used cryotherapy modalities include ice pack and computer-assisted cryotherapy [10]. Despite its widespread use, systematic reviews on the efficacy and safety of cryotherapy post-TKA have shown varied methodologies and outcomes, leading to significant controversies. Some studies suggest the efficacy of cryotherapy remains inconclusive and raise concerns over potential complications such as skin injuries and infections [11].

A reassessment of existing systematic reviews, based on evidence-based practice principles, was conducted to comprehensively synthesize and analyze the available evidence concerning cryotherapy’s effectiveness post-TKA [12]. We employed the A Measure Tool to Assess Systematic Reviews 2 (AMSTAR 2) tool [13], a renowned methodological quality assessment tool for systematic reviews, and the Grading of Recommendations Assessment, Development and Evaluation (GRADE) system [14] to reassess existing systematic reviews.

Although cold therapy is a long-established therapy, recent studies on its use after knee replacement have revealed some emerging applications and potential mechanisms [9]. In addition, the combination of cold therapy with other rehabilitation methods, such as physical therapy, medication, and exercise therapy, provides patients with a multi-modal rehabilitation strategy that may be more effective than monotherapy. In different patient groups, personalized cold therapy applications also show its importance. For example, older patients may have a higher tolerance for cold therapy, while younger patients may require more frequent cold therapy to control postoperative pain [10]. In addition, for patients with specific comorbidities or contraindications to certain medications, a personalized cold therapy regimen can serve as a safe and effective alternative therapy.

To further explore these emerging trends and personalized applications, our study not only reviewed traditional applications of cold therapy, but also incorporated the latest research findings and clinical trials to evaluate its effectiveness and safety in modern rehabilitation practices.

This study aims to clarify the role of cryotherapy in improving rehabilitation outcomes and refining postoperative management strategies for patients who have undergone TKA. By addressing the disparities in previous systematic reviews and offering a clear synthesis of the evidence, this study aims to support informed decision-making in clinical practice. The research followed the PRISMA guidelines.

## 2. Data and methods

### 2.1. Criteria for article inclusion and exclusion

1. Inclusion criteria: ① Study participants: Study participants who underwent TKA without restrictions based on age, gender, race, or nationality. ② Intervention measures: The intervention group was administered cryotherapy, such as traditional ice pack therapy, computer-assisted cryotherapy, either as a standalone treatment or in combination with other treatments such as compression, local nerve block, or continuous passive motion. The control group either received no treatment or utilized non-cryotherapy measures. ③ Outcome indicators: Outcome indicators consisted of pain scores, postoperative analgesic consumption, and other outcomes such as range of motion (ROM), postoperative swelling, and postoperative blood loss. ④ Research type: Systematic reviews and meta-analyses that include randomized controlled trials (RCTs), quasi-experimental studies, or both.
2. Exclusion criteria: ① Systematic review protocols. ② Article not in Chinese or English. ③ Article lacking accessible data. ④ Duplicate publication or article with outdated evidence.

### 2.2. Article retrieval strategy

Retrieval was conducted utilizing electronic databases, including the China Biology Medicine disc (CBM disc), China National Knowledge Infrastructure (CNKI), Wanfang Data, CQVIP database, The Cochrane Library, PubMed, Embase, CINAHL, PEDRO, and Web of Science. Manual searches were conducted to identify relevant research references and potential overlooked documents. Additionally, OpenGrey was utilized to retrieve grey articles. The search strategies for each database were presented in Appendix 1.

The English retrieval keywords used include ‘knee replacement/total knee arthroplasty’, ‘cold therapy/cryotherapy’, and ‘systematic review/meta-analysis’. The Chinese retrieval keywords used include ‘关节置换/关节成形/TKA/TKR (means knee replacement/knee arthroplasty/TKA/TKR in English)’, ‘冰敷/冷疗/冷敷/冷冻疗法/制冷/降温 (means ice pack/cryotherapy/cold compress/cryogenic therapy/refrigeration/cooling in English)’, and ‘系统评价/meta分析 (means systematic review/meta-analysis in English)’.

For instance, in PubMed, the specific retrieval strategies were are as follows: ((Cryotherapy[Mesh]) OR (Cryotherapy[Title/Abstract]) OR (Cryopneumatic[Title/Abstract]) OR (Cryo*[Title/Abstract]) OR (Cold[Title/Abstract]) OR (Cryotherapy[Title/Abstract]) OR (Cold treatment[Title/Abstract]) OR (Ice[Title/Abstract]) OR (Ice Bag*[Title/Abstract]) OR (Ice pack*[Title/Abstract]) OR (Icing[Title/Abstract]) OR (Cooling[Title/Abstract]) OR (Cooling water[Title/Abstract]) OR (Cold Effects[Title/Abstract])) AND ((Arthroplasty, Replacement, Knee[Mesh]) OR (Knee Arthroplasty[Title/Abstract]) OR (Knee Replacement[Title/Abstract])) Filters: Meta-Analysis, Systematic Review, from 2018/1/1-2023/9/15.

The retrieval strategy focused on retrieving the most recent articles from the past five years, using a combination of thematic words and free words. The retrieval was confined to articles published between January 1, 2018, and September 15, 2023, in both Chinese and English languages.

### 2.3. Article screening and data extraction

The initial screening phase was designed to eliminate duplicates and streamline the literature review process, employing EndNote X7, a literature management software. This phase involved identifying overlaps among retrieved articles. In cases where duplicate systematic review articles were found through multiple search criteria, a systematic approach was adopted. This involved prioritizing the retention of the most recent articles, those of the highest methodological quality, and those most pertinent to the research question [15].

Two researchers independently reviewed the titles and abstracts of the retrieved articles. Following this, articles that did not meet the inclusion criteria in the initial phase were excluded. The researchers then conducted a comprehensive review of the full texts of the articles to assess whether they met the inclusion criteria, and subsequently cross-validated the included articles to ensure consensus. Discrepancies were addressed through discussion between the researchers or by seeking the opinion of a third researcher until consensus was reached. The data collection carried out by the two researchers was based on a pre-designed data extraction form. The form consisted of basic information of the study, sample size, intervention methods, outcome indicators, and principal outcomes.

### 2.4. Assessment methods for literature and evidence quality

Two researchers, each systematically trained in evidence-based methods, independently completed the assessment. Upon completion, their findings were compared. Any disparities were resolved through discussion or consultation with a third researcher to reach a consensus.

1. Tools for assessing literature quality: The assessment was carried out using AMSTAR2 [13]. It contains 16 criteria. Each criterion is evaluated as ‘Yes’, ‘No’, ‘or’ ‘Partially yes’. A criterion that fully meets the assessment criteria is labeled as ‘Yes,’ while one that partially meets the criteria is labeled as ‘Partially yes.’ If a criterion lacks relevant information, it is categorized as ‘No.’ The AMSTAR2 research team identified 7 key items crucial for the implementation procedures of systematic reviews and their resultant validity. The 7 key items are items 2, 4, 7, 9, 11, 13, and 15. The validity of systematic review conclusions was classified into four grades: High validity: No deficit or deficits present in only one non-critical area; Intermediate validity: Deficits present in one or more non-critical areas. Low validity: Deficits present in one critical area, with or without deficits in one non-critical area. Exceedingly low validity: Deficits present in more than one critical area, with or without deficits in one non-critical area.
2. Tools for assessing evidence quality: The quality of evidence for each outcome indicators was independently assessed using the GRADE system [16]. The grading considers factors that might downgrade or upgrade the evidence quality. The potential for downgrading is evaluated based on risk of bias (such as improper randomization, unblinded studies, or selective reporting), inconsistency (notable heterogeneity without sufficient explanation), indirectness (irrelevance to the study population), imprecision (wide confidence intervals or small sample sizes), and publication bias (typically studies funded by industry). Conversely, evidence quality may be upgraded based on large effect sizes, the presence of a dose–response gradient, or if biases likely underestimated an effect.

Based on these assessments, evidence related to outcome indicators was categorized into four grades: high quality, low quality, intermediate quality, and exceedingly low quality. Evidence that warrants no downgrading is of high quality, suggesting minimal potential for altering current conclusions through additional research. Evidence requiring a downgrade of one grade is of intermediate quality, suggesting a notable potential for further research to affect the existing findings and possibly change the current conclusion. Evidence necessitating a two-grade downgrade is of low quality, indicating a significant likelihood of further research significantly influencing the existing results and potentially changing the conclusion. Evidence requiring a three-grade downgrade is of exceedingly low quality, signifying a high probability of further research significantly impacting the existing results and potentially altering the conclusion.

## 3. Results

### 3.1. Article screening process and outcomes

**Figure 1.**
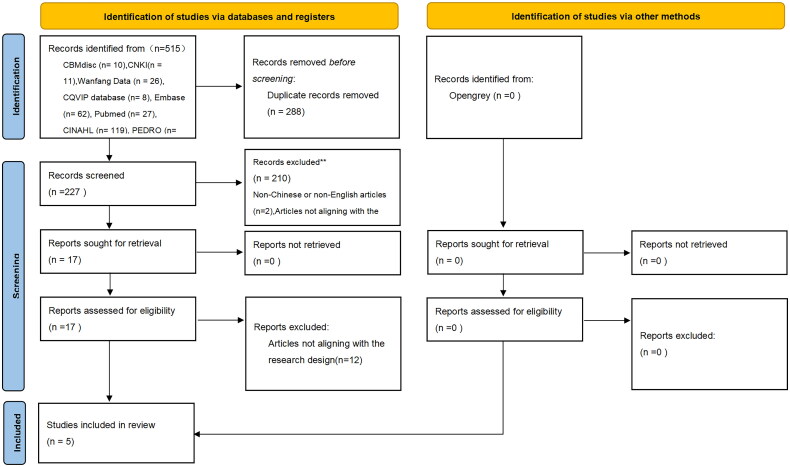
Workflow chart of literature screening.

Initially, 517 articles were identified. Following the exclusion of duplicates and articles incongruent with the research theme or content, a final selection of 5 articles were determined to be suitable for this study. Figure 1 illustrates the detailed article screening workflow.

### 3.2. Basic characteristics of included articles

Among the 5 articles included in this study, 2 [17,18] were in Chinese and 3 [19–21] were in English. The predominant type of studies consisted of randomized controlled trials (RCTs), the number of research indicators included by the institutes ranged from 5 to 22. Table 1 illustrates the fundamental characteristics of the included articles.

**Table 1.** General characteristics of the five included articles.

Publication time and country	Researchers	Type of included original studies	Number of included articles (number) /sample size (case number)	Intervention measures		Outcome indicator	Main conclusions
				Experimental group	Control group		
2019 China	Liu et al. [17]	RCT	10/1070	Cycled cryotherapy, compressive cycled cryotherapy, ice pack, compressive local cryotherapy	Other rehabilitation treatments	Pain, hemoglobin level, dosage of opioids, range of motion (ROM)	Cryotherapy can help alleviate postoperative pain reactions in patients within 2 weeks following surgery and reduce the extent of postoperative hemoglobin reduction. However, it does not significantly improve joint mobility or reduce the use of analgesic medications.
2021 China	Chen et al. [18]	RCT	8/783	Intermittent non-compressive cryotherapy	Non-cryotherapy measures	Pain, postoperative drainage volume of knee joint	Intermittent non-compressive cryotherapy provides analgesic and hemostatic benefits for patients following knee arthroplasty.
2023 Australia	Aggarwal [19]	RCT/Controlled study	22/1839	Cryotherapy either alone or in combination with other treatments (Compression of local nerve tissue or sustained passive movement)	Without treatment or solely using other treatments	Blood loss volume, pain, blood transfusion rate, ROM, knee joint function, number of adverse events, number of cases where cryotherapy was discontinued due to adverse events	The potential benefits of cryotherapy in reducing blood loss, alleviating pain, and enhancing joint mobility are insufficiently demonstrated to justify its use.
2023 Korea	Lee et al. [20]	RCT	8/490	Ice pack, computer-aided cryotherapy	Non-cryotherapy measures	Pain, opioid use, ROM, and patient satisfaction swelling, blood loss volume, ROM, function	Cryotherapy can alleviate pain following TKA, but its effect on swelling is not clear.
2023 Brazil	Krampe et al. [21]	RCT	5/648	Computer-aided cryotherapy, cryotherapy with gradually increasing pressure.	No treatment, using placebos, or simulation therapy	Pain, ROM and function	Cryotherapy can alleviate postoperative pain.

### 3.3. Results of the assessment of methodological quality of included literature

The methodological quality of the 5 articles included in this study was assessed using AMSTAR 2. Among them, 2 articles were rated as exhibiting high methodological quality, while 3 were deemed to exhibit exceedingly low methodological quality (Table 2).

**Table 2.** Assessment results of the quality of the five included systematic review articles.

Included articles	①	②	③	④	⑤	⑥	⑦	⑧	⑨	⑩	⑪	⑫	⑬	⑭	⑮	⑯	Quality grade
Liu et al. [17]	Yes	No	Yes	Yes	Yes	Yes	No	Yes	No	No	Yes	Yes	Yes	Yes	Yes	Yes	Exceedingly low quality
Chen et al. [18]	Yes	No	Yes	Yes	Yes	Yes	No	Yes	Yes	No	Yes	No	No	No	Yes	Yes	Exceedingly low quality
Aggarwal [19]	Yes	Yes	Yes	Yes	Yes	Yes	Yes	Yes	Yes	Yes	Yes	Yes	Yes	Yes	Yes	Yes	High quality
Lee et al. [20]	Yes	Yes	Yes	Yes	Yes	Yes	No	Yes	Yes	No	–	–	Yes	Yes	–	Yes	High quality
Krampe et al. [21]	Yes	No	Yes	Yes	Yes	Yes	No	Yes	Yes	No	Yes	Yes	Yes	Yes	Yes	Yes	Exceedingly low quality

*Note*: – represents the absence of meta-analysis. ① Does the researched issue and inclusion criteria cover all elements of PICO? ② Was the research methodology for systematic review determined prior to implementation? Are there any discrepancies between the report and the study plan proposal? ③ Does the author specify the type of included studies when citing literature? ④ Did the author employ a comprehensive literature retrieval strategy? ⑤ Was the literature screening conducted by two individuals independently? ⑥ Was the data extraction carried out by two individuals independently? ⑦ Does the literature provide a list of excluded studies and reasons for their exclusion? ⑧ Has the author sufficiently described the basic features of articles included in the study? ⑨ Did the author employ appropriate tools to assess the risk of bias in the included studies? ⑩ Did the author report the funding sources for the studies in the systematic review article? ⑪ Has the author employed appropriate statistical methods to consolidate the results of the meta-analysis in the systematic review article?⑫ Has the author considered the risk of bias in the included studies in the systematic review article, as well as potential influences stemming from the amalgamation of other evidence, if a meta-analysis was conducted? ⑬ Did the author consider the risk of bias in the studies included in the systematic review article when explaining/discussing the results of the systematic review? ⑭ Does the author provide an explanation or discussion on the heterogeneity observed in the results of the systematic review? ⑮ Has the author thoroughly investigated publication bias and discussed its potential impact on the research results if quantitative incorporation is conducted? ⑯ Did the author report any potential conflicts of interest, including any funding received for conducting the systematic review?

### 3.4. Results of the quality assessment of outcome indicators from the evidence

A comprehensive evaluation of 18 outcome indicators, analyzed through meta-analysis across the 5 articles, was subjected to evidence quality grading assessment. This assessment was conducted using the GRADE system. A GRADE analysis of the 18 outcome indicators showed that swelling was intermediate-quality evidence (6%, 1/18), hemoglobin, pain score within 24 h after surgery, pain score within 48 h after surgery, pain score within 72 h after surgery, blood loss volume, pain score on the next day of surgery and ROM were low-quality evidence (39%, 7/18), and postoperative pain, dosage of opioids, postoperative mobility, the wound drainage volume within 24 h after surgery, the wound drainage volume within 48 h after surgery, rate of blood transfusion cases, knee joint function, total number of adverse events, the number of withdrawals due to adverse events and pain were exceedingly low-quality evidence (55%, 10/18) (Table 3).

**Table 3.** Assessment results of evidence quality of outcome indicators.

		Number of studies	Risk of bias	Inconsistency	Indirectness	In accuracy	Publication bias	Evidence quality
Liu et al. [17]	Postoperative pain	2	−1^a^	−2^b^	0	0	0	Exceedingly low quality
	Hemoglobin	4	0	−2^b^	0	0	0	Low quality
	Dosage of opioids	2	0	−2^b^	0	−1^e^	0	Exceedingly low quality
	Postoperative mobility	3	0	−2^b^	0	−1^e^	0	Exceedingly low quality
Chen et al. [18]	Pain score within 24 hours after surgery	4	0	−2^b^	0	0	0	Low quality
	Pain score within 48 hours after surgery	4	0	−2^b^	0	0	0	Low quality
	Pain score within 72 hours after surgery	5	0	−2^b^	0	0	0	Low quality
	The wound drainage volume within 24 hours after surgery	3	0	−2^b^	0	−1^e^	0	Exceedingly low quality
	The wound drainage volume within 48 hours after surgery	3	0	−1^c^	0	−1^e^	0	Exceedingly low quality
Aggarwal et al. [19]	Blood loss volume	12	-^a^	−2^b^	0	0	0	Low quality
	Pain score on the next day of surgery	6	−2^a^	0	0	0	0	Low quality
	Rate of blood transfusion cases	2	−1^a^	−2^b^	0	−1^e^	0	Exceedingly low quality
	ROM	3	−1^a^	0	−1^d^	0	0	Low quality
	Knee joint function	4	−1^a^	−2^b^	0	0	0	Exceedingly low quality
	Total number of adverse events	16	−1^a^	0	−1^d^	−1^e^	0	Exceedingly low quality
	The number of withdrawals due to adverse events	19	−1^a^	0	−1^d^	−1^e^	0	Exceedingly low quality
Lee et al. [20]	Swelling	8	−1^a^	0	0	0	0	Middle
Krampe et al. [21]	Pain	5	−1^a^	−2^b^	0	−1^e^	0	Exceedingly low quality

*Note*: ‘–1’ represents downgrading one grade; ‘0’ represents downgrade; ‘–2’ represents downgrading two grades. Among the downgrade reasons, ^a^ represents large risk of bias in random grouping, blind study, undisclosed allocation, completeness of results and data, and partial disclosure of results; ^b^ represents the included studies with 75% ≤ *I*^2^ ≤ 100% and no adequate explanation; ^c^ represents the included studies with 50% ≤ *I*^2^ ≤ 75% and no adequate explanation; ^d^ represents limitations in the measurement of the outcomes of the included studies (diversity in measurement definitions and time); ^e^ represents the sample size of the included studies is too small, either <400 or a wide 95% confidence interval (CI).

## 4. Discussion

The systematic review articles, examined in this study exhibited significant variability in quality. Specifically, two articles[19,20] are rated as exhibiting high methodological quality, while three [17,18,21] are classified as exhibiting exceedingly low in methodological rigor. Among the five articles examined, it was observed that three [17,18,21] lacked a preliminary research plan. Only one article [19] incorporated a list of excluded literature. One article [17] failed to utilize a tool for assessing risk of bias in the included literature. Only one article [19] disclosed the funding source for its primary research. One article [20] excluded the data for merging analysis, and one article [18] failed to discuss about risk of bias and heterogeneity.

These identified deficiencies may compromise the rigor of the research findings and undermine the reliability of the systematic review. To enhance the quality of evidence, researchers should predefine a research plan and register it before conducting any relevant systematic reviews in future studies. Furthermore, they should thoroughly document their retrieval strategy, list all excluded literature, disclose the funding sources of included literature, and provide other pertinent details. Implementing these measures will offer a more comprehensive and significant foundation for evidence.

The existing level of evidence substantiating the efficacy of cryotherapy following TKA necessitates augmentation. Most of the evidence regarding outcome indicators in the articles that were reassessed are classified as either low or exceedingly low according to CRADE grading system. The implementation of a blinding strategy for both medical personnel and patients is challenging due to the specific nature of cryotherapy. Therefore, the absence of a blinding strategy cannot be considered as a primary factor contributing to evidence downgrade. However, certain preliminary studies either failed to incorporate random grouping or lacked comprehensive data reporting, potentially introducing biases into the findings. The specific methods, durations, temperatures, and frequencies of cryotherapy incorporated in various studies exhibit considerable heterogeneity, potentially leading to inconsistencies and lacking directives in the outcomes. Certain outcome indicators are characterized by wide confidence intervals and insufficient supportive sample sizes, potentially indicating inaccuracies in the reported outcomes. The assessment of publication bias is acceptable showing no discernible signs of commercial sponsorship.

The aforementioned conditions result in varying degrees of evidence degradation. Therefore, when utilizing these conclusions to make clinical application decisions and guide clinical practices, practitioners must meticulously assess their efficacy and validity. These assessments should be customized to the individual circumstances of each patient.

### 4.1. Cold treatment tools

In the practice of cold treatment after knee replacement, traditional tools such as ice packs and chemical refrigeration bags are widely used due to their ease of operation and low cost. However, these tools have some limitations, such as the inability to adjust the temperature, the need to change frequently, the surface hardness is not suitable for fitting to the skin, and the possibility of condensation wetting the wound dressing, increasing the risk of infection. Nevertheless, their ease of generalization cannot be ignored. In contrast, modern computer-aided cryotherapy equipment offers significant advantages with its constant temperature control, guaranteed contact surface, and anti-reflux capabilities [11]. The cyclic pressure cold therapy system realizes the heat exchange between the ice pack and the skin through pulse pressure to achieve a lasting cooling effect. In the clinical selection of cold therapy tools, the effect of cold therapy, patient acceptance, recognition and economic cost should be considered comprehensively. It is suggested that future studies should pay more attention to the effectiveness and cost-effectiveness comparison between cold therapy equipment and traditional methods, so as to provide data support for clinical decision-making. Moreover, the use of ice packs serves as a potent adjunctive therapeutic option, due to its straightforward application and economic viability. While in certain studies there has been continuous assessment on computer-aided cryotherapy, existing evidence[22] indicates that this approach fails to deliver significant benefits or demonstrate cost efficiency when compared to conventional ice packs.

### 4.2. Cold therapy parameters

The implementation of cold therapy depends on a series of parameters, including timing, temperature, frequency and duration, but there is no uniform standard. In this study, different parameters of cold therapy were synthesized, and their effects were evaluated and compared. Under the premise of ensuring safety, the time of cold therapy tends to advance, and some evidence shows that cold therapy can start immediately after surgery, or even advance to the operation. With regard to cold treatment temperature, modern equipment can determine and maintain a constant temperature. The duration of cold therapy is also varied, ranging from 2 h to 24 h, and some experts suggest that cold therapy can be extended up to two weeks after surgery to reduce pain reactions [8]. Research indicates that the immediate application of cryotherapy postsurgery is deemed optimal for preventing the propagation of secondary tissue injuries [23]. Cryotherapy is particularly effective at reducing pain intensity during the early postoperative period, especially within the first 48 h following surgery. One possible explanation for this effect is that cryotherapy slows down the neurological pain transmission, thereby alleviating pain [24].

### 4.3. Cold therapy effect

Compared to the control group, patients using cryotherapy reported diminished swelling, which is thought to enhance joint function and reduce pain. This is in line with a systematic review on cryotherapy after acute soft tissue trauma, which corroborated that cryotherapy helps to mitigate postoperative pain and swelling across various procedures including cruciate ligament reconstruction, total knee arthroplasty, total hip arthroplasty, arthroscopic examination, lateral retinaculum release, and carpal tunnel release [25]. Moreover, while long-term studies are necessary to solidify these outcomes, early improvements in joint mobility and functional recovery have been observed in patients undergoing cryotherapy. As indicated by a meta-analysis assessing the effect of cryotherapy on postoperative pain following anterior cruciate ligament reconstruction of the knee, it was discovered that it significantly reduces pain levels. However, its efficacy in improving knee joint mobility was not pronounced [26]. This is not consistent with the findings of this study. One potential reason for this variance could be attributed to variations in the application of cryotherapy. Cryotherapy has demonstrated a potential in decreasing the reliance on analgesics and non-steroidal anti-inflammatory drugs following a wrist or thumb basal joint surgery [27]. Despite these promising results, the limited number of research studies on this topic means that definitive conclusions remain elusive at this stage. Based on the reassessment of five systematic review articles, cryotherapy emerges as a safe non-pharmaceutical intervention for managing pain and swelling. It is particularly beneficial for patients who face limitations or adverse reactions to medication. Most studies have been positive about the effect of cold therapy after knee replacement, but some studies have found no clear benefits, and these differences may be due to a variety of factors, including patient baseline characteristics and specific application of cold therapy (such as temperature, duration, and frequency). The evidence in this study suggests that cold therapy can effectively relieve postoperative pain and promote the resolution of knee swelling. Guidelines from the American Physical Therapy Association [8] also recommend the use of cold therapy for early postoperative pain management and rate its quality of evidence as high and its recommended strength as moderate. Studies have shown [28] that there is a correlation between postoperative deep tissue temperature changes in the surgical area and postoperative joint motion recovery, which provides data support for the application of cold therapy in promoting early recovery.

### 4.4. Study limitations

This research only incorporated Chinese and English ­literature, potentially introducing language and regional biases that constrain the breadth and representativeness of the study. Additionally, there is an imbalance in the age and gender distribution of samples in the literature reviewed. Furthermore, there was notably inconsistency in the inclusion and exclusion criteria across different articles. Such discrepancies can introduce biases into the research findings, affecting the reliability and validity of the conclusions drawn from this study. The absence of PROSPERO registration, while consistent with reassessment study norms, may impact protocol transparency. Future work will prioritize prospective registration.

Therefore, for a comprehensive assessment of the effectiveness and safety of cryotherapy, future studies should incorporate more rigorous research designs, such as multicenter randomized controlled trials with large sample sizes. These measures will improve both the external and internal validity of the research. In future research, it is imperative to account for the individualized nature of patients’ conditions, customize the implementation of cryotherapy to parameters like temperature, duration, and frequency, and consider other variables that may influence efficacy. These variables include baseline health, surgical techniques, and concurrent rehabilitation measures of the patients. Future research should also concurrently focus on the long-term effect of cryotherapy on postoperative rehabilitation, encompassing the restoration of joint function, enhancements in quality of life, and prevention of long-term complications. Moreover, it is crucial to conduct a cost-efficiency analysis to ensure prudent utilization of medical resources. This analysis should evaluate the economic viability and benefits of implementing cryotherapy following TKA.

## 5. Conclusion

Cryotherapy, as a component of physiotherapy, plays an important role in the initial stages of rehabilitation following TKA. By inducing local tissue hypothermia, it effectively slows down cell metabolism and vascular perfusion. Consequently, this diminishes postoperative pain to a certain extent, decreases joint swelling, and aids in improving joint mobility. The results of existing clinical studies exhibit heterogeneity. Furthermore, certain studies exhibit deficiencies in research design and quality, thereby compromising the comprehensiveness in their evidence and their applicability as a significant aid in clinical practice.

To summarize, although cryotherapy following TKA exhibits potential clinical benefits, additional high-quality research is imperative to completely validate its efficacy and safety. This will help encourage wider acceptance of TKA in clinical settings. Improved research efforts will furnish clinical practitioners with a more comprehensive scientific foundation, thereby enabling the administration of increasingly customized and personalized treatment regimens for patients.

## Supplementary Material

PRISMA_2020_checklist.docx

Appendix1.docx

## Data Availability

The datasets used and/or analysed during the current study available from the corresponding author on reasonable request.

## Abbreviations

TKA: Total Knee Arthroplasty
AMSTAR 2: A Measure Tool to Assess Systematic Reviews 2
GRADE: Grades of Recommendations Assessment, Development and Evaluation
ROM: Range of Motion
RCT: Randomized Controlled Trial
