# Supplementary material for: A reassessment of existing systematic reviews evaluating the effectiveness of cryotherapy in patients following total knee arthroplasty
Source: Ann Med. 2025 Jun 4;57(1):2512432. doi: 10.1080/07853890.2025.2512432 (PMC12138932; doi:10.1080/07853890.2025.2512432)
Supplement: Appendix1.docx [file IANN_A_2512432_SM6275.docx]

**Appendix1.** Search strategy.

|  | **Database** | **Search strategy syntax** |
| --- | --- | --- |
| 1 | PubMed | ((Cryotherapy[Mesh]) OR (Cryotherapy[Title/Abstract]) OR (Cryopneumatic[Title/Abstract]) OR (Cryo*[Title/Abstract]) OR (Cold[Title/Abstract]) OR (Cryotherapy[Title/Abstract]) OR (Cold treatment[Title/Abstract]) OR (Ice[Title/Abstract]) OR (Ice Bag*[Title/Abstract]) OR (Ice pack*[Title/Abstract]) OR (Icing[Title/Abstract]) OR (Cooling[Title/Abstract]) OR (Cooling water[Title/Abstract]) OR (Cold Effects[Title/Abstract]))  AND ((Arthroplasty, Replacement, Knee[Mesh]) OR (Knee Arthroplasty[Title/Abstract]) OR (Knee Replacement[Title/Abstract]))  Filters: Meta-Analysis, Systematic Review, from 2018/1/1–2023/9/15. |
| 2 | Cochrane Library | ((MH "Cryotherapy") OR (Cryotherapy:ti,ab,kw) OR (Cryopneumatic:ti,ab,kw) OR (Cryo*:ti,ab,kw) OR (Cold:ti,ab,kw) OR (Cold treatment:ti,ab,kw) OR (Ice:ti,ab,kw) OR (Ice Bag*:ti,ab,kw) OR (Ice pack*:ti,ab,kw) OR (Icing:ti,ab,kw) OR (Cooling:ti,ab,kw) OR ("Cooling water":ti,ab,kw) OR ("Cold Effects":ti,ab,kw))  AND  ((MH "Arthroplasty, Replacement, Knee") OR ("Knee Arthroplasty":ti,ab,kw) OR ("Knee Replacement":ti,ab,kw))  AND (meta-analysis OR "systematic review")  AND  (YEAR >= 2018 AND YEAR <= 2023) |
| 3 | EMBASE | ('cryotherapy'/exp OR 'cryotherapy':ti,ab OR 'cryopneumatic':ti,ab OR 'cryo*':ti,ab OR 'cold':ti,ab OR 'cold treatment':ti,ab OR 'ice':ti,ab OR 'ice bag*':ti,ab OR 'ice pack*':ti,ab OR 'icing':ti,ab OR 'cooling':ti,ab OR 'cooling water':ti,ab OR 'cold effects':ti,ab)  AND  ('knee arthroplasty'/exp OR 'knee replacement'/exp OR 'knee arthroplasty':ti,ab OR 'knee replacement':ti,ab)  AND  ('meta analysis'/de OR 'systematic review'/de)  AND  ([2018-2023]/py) |
| 4 | Web of Science | TS=(Cryotherapy OR Cryopneumatic OR Cryo* OR Cold OR "Cold treatment" OR Ice OR "Ice Bag*" OR "Ice pack*" OR Icing OR Cooling OR "Cooling water" OR "Cold Effects")  AND  TS=("Knee Arthroplasty" OR "Knee Replacement")  AND  TS=("systematic review" OR "meta-analysis")  AND  PY=(2018-2023) |
| 5 | CINAHL (via EBSCOhost) | (MH "Cryotherapy" OR TI Cryotherapy OR AB Cryotherapy OR TI Cryopneumatic OR AB Cryopneumatic OR TI Cryo* OR AB Cryo* OR TI Cold OR AB Cold OR TI "Cold treatment" OR AB "Cold treatment" OR TI Ice OR AB Ice OR TI "Ice Bag*" OR AB "Ice Bag*" OR TI "Ice pack*" OR AB "Ice pack*" OR TI Icing OR AB Icing OR TI Cooling OR AB Cooling OR TI "Cooling water" OR AB "Cooling water" OR TI "Cold Effects" OR AB "Cold Effects")  AND (MH "Arthroplasty, Replacement, Knee" OR TI "Knee Arthroplasty" OR AB "Knee Arthroplasty" OR TI "Knee Replacement" OR AB "Knee Replacement")  AND (PT "Systematic Review" OR PT "Meta Analysis")  AND (2018-2023) |
| 6 | PEDRO | Search Fields: Title & Abstract  Keywords: (cryotherapy OR cryopneumatic OR cryo* OR cold OR "cold treatment" OR ice OR "ice bag" OR "ice pack" OR icing OR cooling OR "cooling water" OR "cold effects")  AND  ("knee arthroplasty" OR "knee replacement")  Filters:  - Limit to "Systematic Reviews"  - Publication Date: 2018-2023 |
| 7 | CNKI | (SU=('冷疗' OR '低温疗法' OR '冰敷' OR '冷敷' OR '冰袋*' OR '冷气雾' OR '冷却' OR '冷效应')  AND  SU=('膝关节置换' OR '全膝关节置换' OR '人工膝关节置换' OR 'TKA' OR '膝关节成形术')  AND  (SU=('系统评价' OR 'Meta分析' OR '荟萃分析'))  AND  (发表时间 2018/1/1–2023/9/15) |
| 8 | Wanfang | (主题:("冷疗法" OR "低温疗法" OR "冰敷" OR "冷敷" OR "冰袋*" OR "冷气雾" OR "冷却" OR "冷效应")  AND  主题:("膝关节置换" OR "全膝关节置换" OR "人工膝关节置换" OR "TKA" OR "膝关节成形术")  AND  (文献类型:("系统评价" OR "Meta分析"))  AND  (年份= 2018-2023) |
| 9 | VIP | (M=('冷疗法' OR '低温疗法' OR '冰敷' OR '冷敷' OR '冰袋' OR '冷气雾') OR T=('冷疗' OR '低温' OR '冷却'))  AND  (M=('膝关节置换' OR '全膝关节置换' OR '人工膝关节置换') OR T=('膝关节成形术' OR '关节置换术'))  AND  (R=('系统评价' OR 'Meta分析'))  AND  (时间范围 >= 2018.1 AND 出版时间 <= 2023.9) |
| 10 | China Biology Medicine disc (CBM disc) | 主题词=(冷疗法 OR 低温疗法) OR 关键词=(冷疗* OR 低温 OR 冰敷 OR 冷敷 OR 冰袋* OR 冷气雾 OR 降温 OR 冷却)  AND  主题词=("关节成形术, 置换, 膝" OR "关节置换") OR 关键词=("膝关节置换" OR "全膝关节置换" OR "人工膝关节置换" OR "膝关节成形术")  AND  (文献类型=Meta分析 OR 文献类型=系统评价)  AND  (年代 >= 2018 AND 年代 <= 2023) |
